# The Burden of Early-Childhood Atopic Disease: Prevalence and Risk Factors for Allergic Rhinitis in an Informal Settlement in South Africa

**DOI:** 10.3390/children13060781

**Published:** 2026-06-04

**Authors:** Velisha Thompson, Joyce Shirinde, Masilu D. Masekameni, Thokozani P. Mbonane

**Affiliations:** 1 Department of Environmental Health, Faculty of Health Sciences, University of Johannesburg, Johannesburg 2001, South Africa; velishat@joburg.org.za (V.T.); maseksd@unisa.ac.za (M.D.M.); 2 School of Health Systems and Public Health, Health Sciences Faculty, University of Pretoria, P.O. Box 667, Pretoria 0001, South Africa; 3 Development Studies, School of Social Sciences, University of South Africa, Pretoria 0003, South Africa

**Keywords:** allergic rhinitis, preschool children, informal settlements, environmental exposures, rhinoconjunctivitis, early-onset atopic disease, indoor air pollution, environmental health, South Africa

## Abstract

**Highlights:**

**What are the main findings?**

- Elevated disease prevalence: The study found that 37.3% of preschool children had allergic rhinitis, and 47.3% experienced general rhinitis symptoms.
- Dominant environmental hazards: Exposure to open sewerage pipelines increased the odds of allergic rhinitis nearly fivefold (AOR: 4.85). Residing near the waterway (AOR: 1.89) and using biomass fuels were also significant risk factors.
- Cumulative residency risk: Children living in the township for more than 24 months were nearly three times more likely (AOR: 2.74) report symptoms of the condition compared to those with less than 12 months of exposure.

**What are the implications of the main findings?**

- Multisectoral intervention requirement: Pharmacological treatments are inadequate for managing the high disease burden; safeguarding pediatric respiratory health in informal settings requires urgent, multisectoral interventions focused on safe sanitation and clean household energy.
- Clinical screening for atopic progression: The connection between allergic rhinitis and conditions like asthma and eczema supports the “atopic march” and “one airway, one disease” concepts, highlighting the need for clinicians in resource-limited settings to screen for allergic rhinitis when a child presents with any atopic condition.
- Environmental policy prioritization: Recognizing macro-environmental hazards, such as open sewage systems and localized outdoor pollutants, as significant risk factors shows that environmental interventions related to sanitation are essential complements to traditional clinical management for reducing early-onset atopy.

**Abstract:**

Background/Objectives: Allergic rhinitis (AR) is increasingly recognized in early childhood; however, its prevalence and environmental determinants within urban informal settlements remain under-researched. This study investigated the prevalence and risk factors associated with allergic rhinitis among children under five years old attending preschools in Alexandra Township, Johannesburg. Methods: A cross-sectional study was conducted involving 3265 children. Data were collected through self-reported surveys with caregivers, designed to assess demographic, clinical, and environmental variables. Multivariate logistic regression analysis was employed to determine adjusted odds ratios (AORs) and *p*-values for potential environmental and clinical risk factors. Results: The findings show a prevalence of 37.3% (n = 1214) for allergic rhinitis, 47.3% (n = 1544) for rhinitis, and 42.7% (n = 1394) for rhinoconjunctivitis. Exposure to open sewerage pipelines was associated with the highest risk (AOR: 4.85, *p* < 0.001), followed by prolonged residence in the township (greater than 24 months; AOR: 2.74, *p* < 0.001) and proximity to local waterways (AOR: 1.89, *p* < 0.001). Additional significant factors included frequent paracetamol consumption and walking to school, while asthma and eczema exhibited an association with the presence of AR. Conclusions: The elevated prevalence of allergic symptoms within this cohort is linked to localized macro-environmental hazards, suggesting that infrastructural challenges in informal urban settings may influence early respiratory health outcomes. Protecting pediatric respiratory health may necessitate multisectoral interventions, with a specific emphasis on ensuring safe sanitation and clean household energy, to complement traditional clinical management in these vulnerable communities.

## 1. Introduction

Allergic rhinitis (AR) is recognized as a chronic inflammatory disorder of the nasal mucosa triggered by immunoglobulin E (IgE) antibodies [1]. Upon exposure to allergens, a Type I hypersensitivity reaction is initiated, characterized by the degranulation of IgE-sensitized mast cells, which release mediators such as histamine and leukotrienes [1,2]. These mediators contribute to immediate symptoms, including nasal itching, sneezing, and rhinorrhea [3]. In early childhood, the pathophysiology of allergic rhinitis is further shaped by the phenomenon known as the “atopic march,” wherein localized inflammation of the upper airway frequently precedes or coexists with the development of bronchial asthma and eczema [4]. Worldwide, allergic rhinitis impacts up to 40% of children, with symptom onset typically occurring as early as ages two to three [5]. In South Africa, the prevalence of allergic rhinitis is substantial, affecting approximately 20–30% of the population; however, its expression within low-income, informal settlements remains inadequately documented, despite the considerable comorbidities associated with inadequately managed pediatric atopy.

Substantial evidence supports the concept that the rising prevalence of allergic diseases is influenced by both genetic susceptibility and environmental exposures [6]. The disease has been associated with a range of risk factors, including sex, housing conditions, socioeconomic status, mold, exposure to environmental air pollution, tobacco and household air pollution, medication, and pollen season, among others [6,7]. While environmental risk factors are extensively documented, the ‘paracetamol hypothesis’ has recently garnered attention, yet it continues to be a topic of considerable clinical debate [8,9]. Emerging evidence indicates that acetaminophen may lead to a depletion of glutathione levels in the respiratory mucosa, potentially skewing the immune response towards a Th2 (allergic) phenotype [10]. Nevertheless, the existing literature exhibits inconsistencies, with several meta-analyses suggesting that the observed association may be confounded by ‘indication bias,’ wherein the drug is administered to address early, non-specific symptoms of respiratory infections rather than directly causing the atopic condition itself [11].

Research conducted in rural regions and under-resourced countries in Africa remains limited; to date, considerable variability exists in prevalence data across study areas [12,13]. In the context of informal settlements, indoor air pollution is exacerbated by specific structural and socioeconomic factors [14,15]. The reliance on biomass fuels for cooking and heating within inadequately ventilated dwellings, whether formally constructed brick structures or informal shacks, results in elevated concentrations of indoor particulate matter [16,17,18]. Recent studies conducted in comparable sub-Saharan African contexts indicate that these localized microenvironments often retain pollutants, contributing to chronic airway irritation that may mimic or intensify allergic sensitization [19]. In South Africa, allergic rhinitis is a prevalent and significant condition across communities, affecting approximately 20–30% of the population [20]. However, identification of risk factors associated with allergic rhinitis amongst vulnerable groups, particularly in low-income settlements, remains under-researched.

Furthermore, the diagnosis of allergic rhinitis during early childhood poses considerable clinical challenges [21,22]. In children under the age of five, differentiating between chronic atopy and the frequent occurrences of viral upper respiratory infections typical of this developmental stage is particularly challenging [22,23]. As a result, numerous epidemiological studies utilize standardized symptom-based questionnaires [9,24,25,26]. Although these instruments are validated for assessing population-level trends, their interpretation necessitates caution due to the potential for overestimating the true prevalence of IgE-mediated allergic rhinitis [21].

The prevalence of allergic diseases is rising among children in sub-Saharan Africa, yet these conditions are poorly documented and managed in under-resourced informal settlements. In Alexandra Township, children under five are exposed to extreme pollutants, including open sewage and biomass fuel combustion. However, research on identifying the environmental and clinical determinants of early-onset atopic disease in this demographic remains lacking. This knowledge gap hinders the development of effective interventions to address critical pediatric environmental health challenges. This study clarifies the burden and distribution of allergic rhinitis in a vulnerable pediatric population in an informal urban setting. By identifying demographic, spatial, and environmental risk factors such as sanitation infrastructure and cumulative residential exposure, this research provides essential data for public health policy. The findings emphasize that clinical management alone is insufficient and highlight the need for multisectoral interventions focused on safe sanitation and clean household energy to protect children’s long-term respiratory and developmental health.

The primary aim of this study was to investigate the prevalence and identify the risk factors associated with allergic rhinitis and related atopic symptoms among children under five years of age attending preschools in Alexandra Township, Johannesburg. To accomplish this, the study objectives were as follows: (i) assessing the prevalence of allergic rhinitis, rhinitis, and rhinoconjunctivitis using caregiver-reported International Study of Asthma and Allergies in Childhood (ISAAC) questionnaires; (ii) evaluating the associations between demographic and clinical factors, including age, family history, and comorbidities, and the presence of respiratory symptoms; (iii) identifying significant environmental and household risk factors, such as proximity to open sewage, waterways, and the use of biomass fuel, that contribute to the disease burden.

## 2. Materials and Methods

### 2.1. Study Design and Setting

A cross-sectional epidemiological study was conducted from April 2024 to June 2025 in preschools in an informal urban setting (Alexandra Township, City of Johannesburg, Gauteng, South Africa. Alexandra Township is a densely populated urban area located between latitudes 26°05′00.9″ S and 26°06′59.9″ S and longitudes 28°05′08.8″ E and 28°06′15.2″ E [16]. The township’s environmental profile is influenced by the Jukskei River, which flows through the overpopulated settlement [17]. The river serves as a catchment for effluent from surrounding industries and runoff from illegal businesses, uncontrolled waste dumps, and localized agricultural practices, creating a unique microenvironment of concentrated pollutants.

### 2.2. Study Population, Inclusion Criteria and Sample Selection

A list of 161 preschools was provided by the Gauteng Department of Education. From this list, a total of 61 preschools were randomly selected to participate. The study population consisted of children below the age of five years who attended preschool and were residents of the township. The sample size was subsequently calculated using Epi Info 7.2; however, we aimed to target a population of 3000 or more to adhere to the recommendations outlined in the ISAAC manual [18,19]. The study population consisted of 3265 children.

### 2.3. Study Health Outcomes

The study health outcomes (primary and secondary) were measured using caregiver self-reported data from the written ISAAC questionnaire, completed by the children’s parents or guardians. The primary outcome was allergic rhinitis, defined by a “Yes” response to the question: “Has your child ever had hay fever?” It is crucial to note that this outcome relies solely on terminology reported by caregivers through the ISAAC tool, rather than being validated by a physician’s diagnosis or objective clinical assessments.

Secondary outcomes included caregiver self-reported rhinitis and rhinoconjunctivitis, which were identified through the following questions:

- Rhinitis: “Has your child ever had a problem with sneezing, or a runny or blocked nose, when he/she did NOT have a cold or flu?”
- Rhinoconjunctivitis: “In the past, has your child had a nose problem accompanied by itchy, watery eyes?”

### 2.4. Risk Factors in the Study

Two sections were included in the questionnaire. One section for the sociodemographic characteristics of the participants, which included the following: child’s sex (male/female), child’s age (12 months or younger/13–36 months/37 months or older), duration of township stay (less than 12 months/12–24 months/more than 24 months), family asthma history (no/yes), mother or female guardian smoking status (no/yes). This section also asked the following questions: Has the child been experienced or reported to have asthma? (no/yes); Has the child been experienced or reported to have eczema? (no/yes); Has the child been experienced or reported to have food allergy? (no/yes); How often does the child consume takeaways? (never/1–2 times a week/3 or more times a week); How often does the child use pain medication [such as aspirin/paracetamol]? (never/occasionally/frequently). The second section included household and environmental risk factors: household crowding index (HCI) (less than 1 person/1–2 persons/more than 2 persons), residing near a sewerage open pipeline/s (no/yes), exposure to environmental smoking (not exposed/exposed), mode of transport to school (walking/family car/public transport), house structural type (shack/brick) and household energy (coal or wood/electricity).

### 2.5. Data Management and Statistical Analysis

Primary data were cleaned and coded in Microsoft Excel before being exported to the latest version 18 of STATA software (StataCorp, College Station, TX, USA) for comprehensive analysis. Descriptive statistics, including frequencies and percentages, were used to delineate the sociodemographic and clinical characteristics of the study population. To examine the distribution of risk factors, the population was divided based on the presence or absence of the primary health outcome (allergic rhinitis) and secondary outcomes (rhinitis and rhinoconjunctivitis). To identify specific risk factors for allergic rhinitis, a structured two-stage modelling approach was implemented. Initially, a bivariate binary logistic regression analysis was conducted to assess the relationship between allergic rhinitis and all sociodemographic, environmental, and household variables, facilitating the calculation of crude odds ratios. Variables showing a statistically significant difference in the bivariate analysis were then included in a multivariate logistic regression model to determine adjusted odds ratios (AOR). The potential for collinearity among environmental variables was assessed using the Variance Inflation Factor (VIF) to ensure model stability, while missing data were addressed through pairwise deletion, predicated on the assumption of being missing at random. Significance levels were established at *p* < 0.05 using two-tailed tests to evaluate the likelihood of health consequences, thereby ensuring a robust identification of factors influencing upper airway inflammation within this specific geographic context.

### 2.6. Ethical Considerations

The parents of participating children provided the required written informed consent. Permission to conduct this study was obtained from the Gauteng Department of Education, the jurisdiction of preschools. This study received ethical clearance from the Faculty of Health Sciences Research Ethics Committee (REC) of the University of Johannesburg (REC-2662-2024).

## 3. Results

### 3.1. Prevalence of Allergic Rhinitis and Related Symptoms

**Figure 1:**
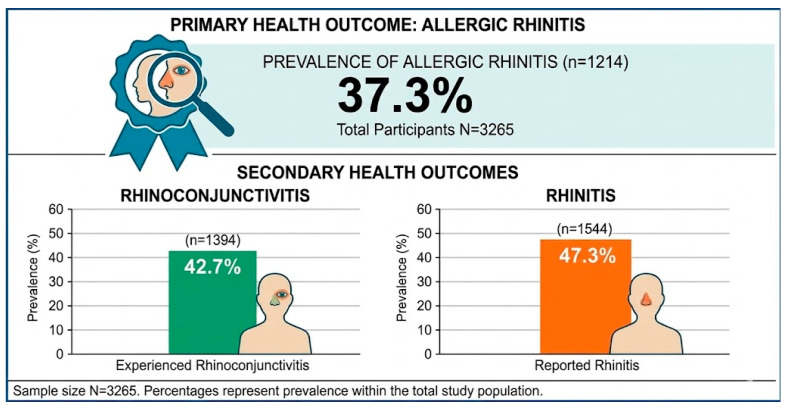
Prevalence of allergic rhinitis, rhinoconjunctivitis and rhinitis.

The prevalence of allergic rhinitis in the study was 37.7% (n = 1214), as shown in Figure 1. Additionally, 1394 participants (42.7%) experienced rhinoconjunctivitis, while 1544 participants (47.3%) reported rhinitis.

### 3.2. Children’s Sociodemographic Factors in the Study

Table 1 presents a comprehensive sociodemographic and clinical profile of the study population. Among the 3265 participants, females marginally outnumber males (52.9% vs. 47.1%), with the majority of the cohort being aged one year or older. A significant trend observed in the data is the robust association between allergic conditions and hereditary factors; for instance, a family history of asthma is reported in 57.8% of individuals with allergic rhinitis, compared to only 32% among those without the condition. Comorbidities are also prevalent, as children reported to have allergic rhinitis, rhinitis, or rhinoconjunctivitis show higher rates of concurrent asthma and eczema. Furthermore, children who have resided in Alexandra Township for over 24 months demonstrate a higher prevalence of allergic rhinitis (47.1%) than their counterparts with shorter residency, suggesting a potential cumulative effect of local environmental exposures. Interestingly, while proximity to a waterway is a contributing factor for approximately one-quarter of the affected children, it does not reveal a significant percentage difference when compared to the healthy cohort. A notable clinical correlation identified pertains to medication usage, where the frequency of paracetamol use is observed to be nearly five times more prevalent in children with allergic rhinitis (22.6%) than in those without (4.9%). Conversely, maternal smoking and the consumption of takeaway food exhibit minimal variance between the groups, indicating that genetic predisposition and the duration of local environmental exposure may be more influential factors in the manifestation of these respiratory conditions within this specific population.

### 3.3. Environmental Risk Factors Related to Allergic Rhinitis in the Study

The physical environment demonstrated significant contrasts between children reported to have allergic rhinitis and those without the condition. The most notable divergence pertained to sanitation: 63.4% of children with allergic rhinitis resided in proximity to open sewerage pipelines or manholes, in contrast to only 17.7% within the healthy cohort. Similarly, a significant majority (87.6%) of children with allergic rhinitis utilized walking as their primary mode of transport to school, whereas those commuting by family car or public transport demonstrated markedly lower rates of the condition. Although the majority of the overall study population had access to electricity and resided in brick houses, comparative data indicate that these structural factors did not confer protective benefits in this particular context. Specifically, children with allergic rhinitis were more frequently found in brick dwellings (91.3%) compared to their healthy counterparts (81.9%), suggesting a potential entrapment of indoor pollutants. Furthermore, reliance on coal or wood for energy showed a higher frequency in children with rhinitis symptoms (8.7%) than in the healthy cohort, further distinguishing the two groups based on household energy exposure. Exposure to environmental smoke was also more common among affected children (28.2%) than those without allergic rhinitis (25.7%), although this difference was less pronounced than the observed macro-environmental sanitation hazards. Table 2 shows the distribution of environmental risk factors discussed above.

### 3.4. Risk Factors Associated with Allergic Rhinitis in This Study

The multivariate logistic regression analysis shown in Table 3 identifies several critical environmental and clinical risk factors affecting the study population, most notably the presence of open sewerage pipelines, which increases the odds of the condition by a factor of 4.85 (*p* < 0.001). Residential history in Alexandra Township demonstrates a clear cumulative effect, wherein residing for more than 24 months nearly triples the risk (AOR 2.74) compared to individuals who have lived there for less than a year. Proximity waterway (AOR 1.63) and the use of coal or wood for energy also emerge as significant environmental factors. From a clinical standpoint, a family history of asthma (AOR 2.05) and the presence of comorbid conditions such as eczema (AOR 1.86) and asthma (AOR 1.49) are strong predictors. Additionally, frequent paracetamol use is associated with a heightened risk (AOR 2.65), while dietary habits also play a role, with individuals consuming takeaway meals three or more times per week exhibiting a 1.54 times higher likelihood of experiencing allergic symptoms. Notably, the data reveals significant protective factors and some unexpected non-significance in traditionally recognized risk areas. Specifically, being older than one year is highly protective compared to infancy, with children over the age of three being 88% less likely to have the condition. Transportation methods also influence outcomes; children who utilize a family car (AOR 0.42) or public transport (AOR 0.48) showed lower odds than those who walk, likely due to reduced exposure to ambient pollutants. Conversely, factors commonly associated with respiratory issues, such as maternal smoking, environmental smoke exposure, and household crowding, did not achieve statistical significance in this specific model (*p* > 0.05). This finding suggests that, within this particular geographic context, structural environmental hazards such as sanitation and long-term residency are more dominant factors of health outcomes than household-level smoking or crowding. The transition from bivariate to multivariable analysis revealed significant changes in the influence of several risk factors. In the bivariate analysis, proximity to waterway and occasional paracetamol use appeared to have a protective or negligible effect. However, in the final multivariable model, after adjusting for open sewerage pipelines and the protective effect of older age, these variables became significant risks. Proximity to waterway shifted from a crude odds ratio (COR) of 0.78 to an adjusted odds ratio (AOR) of 1.64 (*p* < 0.001), indicating that the river’s impact is masked by other socioeconomic and infrastructural factors until those variables are controlled.

## 4. Discussion

This study investigated the risk factors associated with allergic rhinitis among preschool-age children in an informal urban setting in Johannesburg, specifically focusing on Alexandra Township. The results reveal a complex interplay of environmental, clinical, lifestyle, and spatial factors associated with the prevalence of allergic rhinitis in this vulnerable demographic. Notably, the study found a high burden of upper airway inflammatory conditions, with 47.3% of the cohort experiencing rhinitis, 42.7% reporting rhinoconjunctivitis, and 37.3% had allergic rhinitis. These figures are particularly striking given that the study population exclusively comprises children under five years old.

The study findings indicated that the physical environment plays a role or risk factor in the development of attacks related to allergic rhinitis. In this study, exposure to open sewerage pipelines presented the highest risk of any variable analysed, increasing the odds of allergic rhinitis nearly fivefold (AOR: 4.85, *p* < 0.001). Open sewerage is a major source of bioaerosols, endotoxins, and microbial pathogens [20]. Chronic inhalation of these irritants is likely associated with severe airway inflammation, exacerbating or triggering allergic rhinitis symptoms [22].

Similarly, residing in proximity to a waterway increased the association odds with allergic rhinitis (AOR: 1.63, *p* < 0.001). Informal dwellings near urban rivers are frequently subject to dampness, poor drainage, and subsequent mold growth [23,24]. Mold spores are well-documented aeroallergens and are considered a trigger for allergy attacks or reactions [25,26,27]. Furthermore, the duration of exposure to this environment demonstrated a clear dose–response relationship. Compared to children living in the township for less than 12 months, those residing there for 12–24 months (AOR: 1.42, *p* = 0.006) and more than 24 months (AOR: 2.74, *p* < 0.001) showed progressively higher risks. This suggests that cumulative exposure to the specific microenvironment of this informal settlement is associated with the reported allergic symptoms. Reliance on coal or wood for household energy was also identified as a significant risk factor (*p* = 0.006). Biomass fuel combustion releases high levels of indoor particulate matter and volatile organic compounds, which damage the respiratory epithelium and act as adjuvants for allergic sensitization [28,29].

The associations identified between specific household characteristics and the risk of allergic rhinitis necessitate careful interpretation, as they may reflect complex underlying confounding factors rather than direct causal relationships. For example, the increased odds of allergic rhinitis observed in children living in brick houses compared to those in shacks (AOR: 1.39) appear counterintuitive. Rather than the building material itself serving as a risk factor, this association may function as a proxy for diminished natural ventilation and the consequent accumulation of indoor pollutants, moisture, and aeroallergens such as dust mites and mold. Similarly, the high prevalence of symptoms among households utilizing electricity indicates that, even in electrified homes, the surrounding macro-environment or the concurrent use of supplemental biomass fuels for heating may be more significant contributors to respiratory inflammation [30,31,32]. Furthermore, while walking to school was identified as a significant risk factor compared to using family transportation (AOR: 0.42 for family cars), this likely serves as an indicator of cumulative outdoor exposure. Children who walk in Alexandra Township endure prolonged contact with unpaved road dust, vehicle exhaust, and ambient particulate matter from neighborhood open fires [33,34,35]. Therefore, these findings should be regarded as indicators of a child’s total “environmental load” rather than isolated triggers. Recognizing these complexities mitigates the risk of overinterpreting specific variables. As noted, the associations related to housing structural types, electricity consumption, and commuting patterns are likely indicative of residual socioeconomic factors or broader environmental confounding, rather than direct explanatory relationships. This reinforces the necessity for a nuanced understanding.

The study findings align with the “atopic march” hypothesis [4]. A family history of asthma doubled the odds of a child experiencing allergic rhinitis (AOR: 2.05, *p* < 0.001), highlighting the strong genetic predisposition to atopy. Furthermore, children with concurrent atopic diseases showed an elevated association with AR, including asthma (AOR: 1.49, *p* = 0.001), eczema (AOR: 1.86, *p* < 0.001), and food allergies (AOR: 1.25, *p* = 0.041). These findings support the “one airway, one disease” concept, reinforcing the need for clinicians in resource-limited settings to screen for allergic rhinitis when a child presents with any other atopic condition [4,36,37].

Frequent consumption of takeaway meals (3 or more times a week) was associated with allergic rhinitis (AOR: 1.54, *p* < 0.001). Such diets are high in processed fast foods, are typically rich in saturated fats, trans fats, and sodium, and are low in antioxidants [38,39]. This dietary pattern is known to promote systemic inflammation and has been linked to increased prevalence of asthma and rhinitis in global pediatric cohorts [40,41].

Additionally, a relationship was observed with paracetamol (acetaminophen) use. Occasional use (AOR: 1.33, *p* < 0.001) and frequent use (AOR: 2.65, *p* < 0.001) increased the association with allergic rhinitis compared to non-use. This supports the “paracetamol hypothesis,” which suggests that the drug depletes glutathione levels in the respiratory mucosa, reducing antioxidant defenses, increasing oxidative stress, and skewing the immune system toward a Th2 (allergic) phenotype [42].

The data revealed a protective effect for older age groups compared to infants under one year old (1–3 years: AOR: 0.14, *p* < 0.001; >3 years: AOR: 0.12, *p* < 0.001). This finding warrants careful interpretation. It may reflect a survivor bias, differences in parental reporting (infant “snuffles” vs. true AR), or the possibility that the heaviest burden of severe, symptomatic respiratory inflammation in this specific heavily polluted environment occurs during early infancy when airways are smallest and immune systems are most immature.

Notably, factors such as gender, house crowding index, and exposure to environmental tobacco smoke (both maternal prenatal and general environmental) did not reach statistical significance in this model. In the context of Alexandra Township, it is highly possible that the inflammatory impact of macro-environmental pollutants, such as open sewage, biomass fuels, and heavy outdoor dust, overshadows the localized effects of passive smoking or household crowding.

### Strengths and Limitations

A primary limitation of this study is the exclusive reliance on caregiver-reported surveys using the ISAAC questionnaire, without clinical confirmation or objective diagnostic testing, such as skin prick tests or serum IgE measurements. This methodology introduces potential recall bias and variations in caregiver interpretation of terminology, such as “hay fever.” Moreover, in preschool-aged children, there is significant symptom overlap between allergic rhinitis and frequent, non-allergic viral upper respiratory tract infections. The lack of objective testing and physician diagnosis increases the likelihood of symptom misclassification, potentially leading to an overestimation of the true prevalence of IgE-mediated allergic rhinitis in this cohort. Consequently, these findings should be interpreted cautiously as reflecting caregiver-reported upper airway symptomatic inflammation rather than definitive clinical diagnoses.

Thus, these findings likely reflect overall symptomatic upper airway inflammation rather than a strict diagnosis of allergic rhinitis. Temporal and statistical complexities also influence results. Data collection spanned from April 2024 to June 2025, with peak symptoms coinciding with the South African winter, potentially inflating prevalence due to seasonal viral loads and increased biomass fuel use. Additionally, shifts in variables like waterway proximity and diet between bivariate and multivariable models suggest that certain risks were initially obscured by macro-environmental hazards. Despite robust statistical adjustments, residual confounding from unmeasured socioeconomic stressors and exposure misclassification regarding perceived distances remains a concern. Nonetheless, this methodology is a valuable tool for identifying critical environmental determinants of pediatric respiratory distress in under-resourced settings.

## 5. Conclusions

This study identifies a significant burden of pediatric respiratory and allergic symptoms within an informal settlement in Johannesburg, suggesting potential environmental health challenges. The findings indicate that macro-environmental factors, particularly inadequate sanitation and proximity to open sewage, are associated with a heightened prevalence of early-onset allergic rhinitis among preschool children. Moreover, indoor air quality determinants, such as the utilization of biomass fuels, alongside outdoor exposures during daily commutes, appear to contribute to the overall respiratory risk. Although these associations do not establish direct causality, the elevated frequency of symptoms in accordance with the “atopic march” hypothesis highlights an urgent need for further investigation. Addressing these risks may necessitate multisectoral infrastructural interventions aimed at improving safe sanitation and promoting clean household energy solutions to reduce the toxic environmental burden on this vulnerable population.

## Figures and Tables

**Table 1:** Distribution of sociodemographic data according to allergic rhinitis, rhinitis and rhinoconjunctivitis (N = 3.265).

Sociodemographic Data	Total N (%)	Allergic Rhinitis (Yes)	Rhinitis (Yes)	Rhinoconjunctivitis (Yes)
Gender				
Boys	1538 (47.1%)	596 (49.1%)	766 (49.6%)	715 (51.3%)
Girls	1727 (52.9%)	618 (50.9%)	778 (50.4%)	679 (48.7%)
Age				
Younger than 1 year old	303 (9.3%)	223 (18.4%)	212 (13.7%)	226 (16.2%)
1–3 years old	1478 (45.3%)	451 (37.2%)	632 (40.9%)	534 (38.3%)
Older than 3 years old	1484 (45.4%)	540 (44.4%)	700 (45.3%)	634 (45.5%)
Length of stay in Alexandra Township				
Less than 12 months	1394 (42.7%)	380 (31.3%)	514 (33.3%)	464 (33.3%)
12–24 months	630 (19.3%)	262 (21.6%)	386 (25%)	312 (22.4%)
More than 24 months	1241 (38%)	572 (47.1%)	644 (41.7%)	618 (44.3%)
Proximity to Waterway				
No	2382 (73.2%)	926 (76.3%)	1172 (75.9%)	1058 (75.9%)
Yes	883 (26.8%)	288 (23.7%)	372 (24.1%)	336 (24.1%)
Family history of Asthma				
No	1907 (58.4%)	512 (42.2%)	624 (40.4%)	564 (40.5%)
Yes	1358 (41.6%)	702 (57.8%)	920 (59.6%)	830 (59.5%)
Asthma				
No	2693 (82.5%)	930 (76.6%)	1174 (76%)	1064 (76.3)
Yes	572 (17.5%)	284 (23.4%)	370 (24%)	330 (23.7%)
Eczema				
No	2579 (79%)	808 (66.7%)	1102 (71.4%)	986 (70.7%)
Yes	686 (21%)	406 (33.4%)	442 (64.4%)	408 (29.4%)
Food Allergy				
No	2532 (77.5%)	914 (75.3%)	1235 (80%)	1099 (78.8%)
Yes	733 (22.5%	300 (24.7%)	309 (20%)	295 (21.2%)
Takeaway				
Never	1326 (40.6%)	558 (45.9%)	668 (43.3%)	604 (43.3%)
1–2 times a week	1491 (45.8%)	558 (45.9%)	710 (46%)	644 (46.2%)
3 or more times a week	448 (13.7%)	98 (8.2%)	166 (10.7%)	146 (10.5%)
Mother smoked within 1 month of birth				
No	2855 (87.4%)	1064 (87.6%)	1350 (87.4%)	1226 (87.9%)
Yes	410 (12.6%)	150 (12.4%)	194 (12.6%)	168 (12.1%)
Frequency of Paracetamol Use				
Never	2628 (80.5%)	952 (73%)	1144 (74.1%)	1058 (75.9%)
Occasionally	263 (8.1%)	68 (5.2%)	92 (6%)	60 (4.3%)
Frequently	374 (11.4%)	284 (21.8%)	308 (19.9%)	276 (19.8%)

**Table 2:** Environmental risk factors in the study.

Environmental Risk Factor	Total N (%)	Allergic Rhinitis (Yes)	Rhinitis (Yes)	Rhinoconjunctivitis (Yes)
House crowding index				
Less than 1 person	1717 (52.6%)	722 (55.4%)	848 (54.9%)	775 (52.6%)
1–2 persons	1223 (37.5%)	475 (36.4%)	564 (36.5%)	510 (36.6%)
More than 2 persons	325 (10%)	107 (8.2%)	132 (8.6%)	109 (7.8%)
Sewerage open pipeline/s				
No	2131 (65.3%)	444 (36.6%)	678 (43.9%)	574 (41.2%)
Yes	1134 (34.7%)	770 (63.4%)	866 (56.1%)	820 (58.8%)
Exposure to environmental smoking				
Not exposed	2395 (73.4%)	872 (71.8%)	1130 (73.2%)	1012 (72.6%)
Exposed	870 (26.6%)	342 (28.2%)	414 (26.8%)	382 (27.4%)
Mode of transport to school				
Walking	2551 (78.1%)	1064 (87.6%)	1306 (84.6%)	1222 (87.7%)
Family car	324 (9.9%)	62 (5.1%)	118 (7.6%)	84 (6%)
Public transport	390 (11.9%)	88 (7.3%)	120 (7.8%)	88 (6.31)
Housing Structural Type				
Shack	478 (14.6%)	106 (8.7%)	164 (10.6%)	132 (9.5%)
Brick	2787 (85.4%)	1108(91.3%)	1380 (89.4%)	1262 (90.5%)
Household energy				
Coal or wood	271 (8.3%)	100 (8.2%)	134 (8.7%)	106 (7.6%)
Electricity	2994 (91.7%)	1114 (91.8%)	1410 (91.3%)	1288 (92.4%)

**Table 3:** Bivariate and multivariable logistic regression identifying risk factors associated with allergic rhinitis.

Risk Factors	Category	COR (SE)	95%CI	*p*-Value	AOR (SE)	95%CI	*p*-Value
Gender	Boy	Ref					
	Girls	0.98 (0.06)	0.86–1.09	0.080	0.88 (0.08)	0.73–1.05	0.152
Age	Younger than 1 year-old	Ref					
	1–3 years-old	0.16 (0.02)	0.12–0.21	<0.001 *	0.14 (0.02)	0.10–0.20	<0.001 *
	Older than 3 years old	0.21 (0.03)	0.16–0.27	<0.001 *	0.12 (0.02)	0.09–0.17	<0.001 *
Length of stay in Alexandra Township	Less than 12 months	Ref					
	12–24 months	1.90 (0.19)	1.56–2.31	<0.001 *	1.42 (0.18)	1.10–1.83	0.006 *
	More than 24 months	2.28 (0.19)	1.94–2.68	<0.001 *	2.74 (0.33)	2.17–3.46	<0.001 *
Proximity to Waterway	No	Ref					
	Yes	0.76 (0.06)	0.66–0.91	0.002 *	1.89 (1.07)	1.81–1.98	<0.001 *
Family history of Asthma	No	Ref					
	Yes	2.92 (0.22)	2.52–3.38	<0.001 *	2.05 (0.20)	1.70–2.47	<0.001 *
Asthma	No	Ref					
	Yes	2.15 (0.20)	1.79–2.58	<0.001 *	1.49 (0.18)	1.18–1.88	0.001 *
Eczema	No	Ref					
	Yes	3.18 (0.28)	2.67–3.78	<0.001 *	1.86 (0.22)	1.48–2.34	<0.001 *
Food Allergy	No	Ref					
	Yes	1.23 (0.11)	1.04–1.45	0.017 *	1.25 (0.14)	1.01–1.56	0.041 *
Mother smoked within 1 month of birth	No	Ref					
	Yes	0.97 (0.11)	0.78–1.20	0.789	0.96 (0.17)	0.67–1.37	0.901
Takeaway	Never	Ref					
	1–2 times a week	1.82 (0.06)	1.71–1.96	0.012 *	1.92 (0.67)	0.97–3.81	0.063
	3 or more times a week	1.39 (0.05)	1.30–1.49	<0.001 *	1.54 (0.08)	1.40–1.73	<0.001 *
Frequency of Paracetamol Use	Never	Ref					
	Occasionally	0.46 (0.07)	0.34–0.62	<0.001 *	0.33 (0.16)	0.13–0.85	<0.001 *
	Frequently	5.35 (0.66)	4.20–6.82	<0.001 *	2.65 (0.41)	1.96–3.57	<0.001 *
House crowding index	Less than 1 person					Ref	
	1–2 persons	0.84 (0.07)	0.72–0.98	0.026 *	1.02 (0.10)	0.84–1.24	0.845
	More than 2 persons	0.75 (0.10)	0.58–0.96	0.022 *	1.05 (0.18)	0.75–1.46	0.792
Sewerage open pipeline/s	No					Ref	
	Yes	8.04 (0.67)	6.83–9.46	<0.001 *	4.85 (0.46)	4.02–5.84	<0.001 *
Exposure to environmental smoking	Not exposed	Ref					
	Exposed	1.13 (0.09)	0.96–1.33	0.130	1.02 (0.14)	0.78–1.33	0.901
Mode of transport to school	Walking	Ref					
	Family car	0.33 (0.05)	0.25–0.44	<0.001 *	0.42 (0.07)	0.30–0.59	<0.001 *
	Public transport	0.41 (0.05)	0.32–0.52	<0.001 *	0.48 (0.07)	0.36–0.65	<0.001 *
Housing Structural Type	Shack	Ref					
	Brick	2.32 (0.27)	1.84–2.91	<0.001	1.39 (0.22)	1.02–1.89	0.035 *
Household energy	Electricity	Ref					
	Coal or wood	0.99 (0.03)	0.76–1.28	<0.001 *	1.63 (0.17)	1.17–2.75	0.006 *

AOR—adjusted odds ratio; SE—standard error; * shows a statistically significant *p*-value.

## Data Availability

The original contributions presented in this study are included in the article. Further inquiries can be directed to the corresponding authors, and the request must be made in accordance with the Protection of Personal Information Act 4 of 2013.

## Abbreviations

The following abbreviations are used in this manuscript:

AOR	adjusted odds ratio
CI	confidence interval
HCI	household crowding index
HDC	Higher Degree Committee
ISAAC	International Study of Asthma and Allergies in Childhood
REC	Research Ethics Committee
SE	standard error
